# Trained innate immunity modulates osteoblast and osteoclast differentiation

**DOI:** 10.1007/s12015-024-10711-9

**Published:** 2024-03-13

**Authors:** N. R. Rahmani, R. Belluomo, M. C. Kruyt, D. Gawlitta, L. A. B. Joosten, H. Weinans, M. Croes

**Affiliations:** 1 Department of Orthopedics, University Medical Center Utrecht, Utrecht, the Netherlands; 2 Regenerative Medicine Center Utrecht, Utrecht University, Utrecht, the Netherlands; 3 Department of Developmental Biomedical Engineering, Twente University, Enschede, the Netherlands; 4 Department of Oral and Maxillofacial Surgery, Prosthodontics and Special Dental Care, University Medical Center Utrecht, Utrecht, the Netherlands; 5 Department of Internal Medicine, Radboud University Medical Center, Nijmegen, the Netherlands; 6 Department of Medical Genetics, Iuliu Hatieganu University of Medicine and Pharmacy, Cluj-Napoca, Romania; 7 Department of Biomechanical Engineering, Technical University Delft, Delft, the Netherlands

**Keywords:** Innate immune memory, Osteoimmunology, Macrophage, Bone regeneration, MSC

## Abstract

Macrophages are key regulators in bone repair and regeneration. Recent studies have shown that long-term epigenetic changes and metabolic shifts occur during specific immune training of macrophages that affect their functional state, resulting in heightened (trained) or reduced (tolerant) responses upon exposure to a second stimulus. This is known as innate immune memory. Here, we study the impact of macrophages’ memory trait on osteoblast differentiation of human mesenchymal stromal cells (hMSCs) and osteoclast differentiation. An in vitro trained immunity protocol of monocyte-derived macrophages was employed using inactivated *Candida albicans* and Bacillus Calmette–Guérin (BCG) to induce a ‘trained’ state and Pam3CSK4 (PAM) and Lipopolysaccharides (LPS) to induce a ‘tolerance’ state. Macrophages were subsequently cocultured with hMSCs undergoing osteogenic differentiation during either resting (unstimulated) or inflammatory conditions (restimulated with LPS). Alkaline phosphatase activity, mineralization, and cytokine levels (TNF, IL-6, oncostatin M and SDF-1α) were measured. In addition, macrophages underwent osteoclast differentiation. Our findings show that trained and tolerized macrophages induced opposing results. Under resting conditions, BCG-trained macrophages enhanced ALP levels (threefold), while under inflammatory conditions this was found in the LPS-tolerized macrophages (fourfold). Coculture of hMSCs with trained macrophages showed mineralization while tolerized macrophages inhibited the process under both resting and inflammatory conditions. While osteoclast differentiation was not affected in trained-macrophages, this ability was significantly loss in tolerized ones. This study further confirms the intricate cross talk between immune cells and bone cells, highlighting the need to consider this interaction in the development of personalized approaches for bone regenerative medicine.

**Graphical Abstract:**

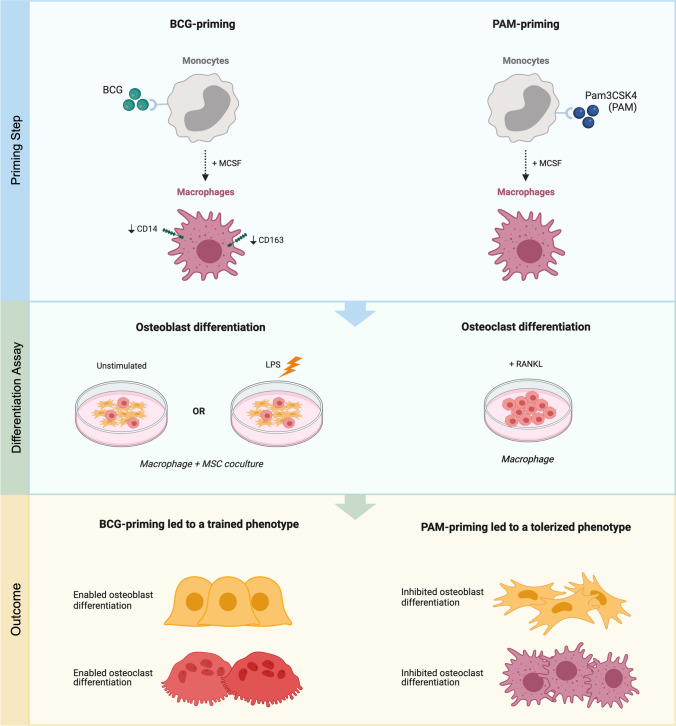

**Supplementary Information:**

The online version contains supplementary material available at 10.1007/s12015-024-10711-9.

## Introduction

Bone repair relies strongly on a well-orchestrated immune response. A recent approach in bone regenerative medicine is rendering bone biomaterials with osteo-immunomodulatory properties, that is, functionalities that instruct the behavior of immune cells in favor of bone formation [1]. The need for appropriate osteo-immunomodulation is derived from the observation that immune and skeletal cells (osteoclasts) share common precursor cells and form an integrated part in bone homeostasis, repair, and disease [2, 3]. The innate immune compartment provides the necessary cytokines and growth factors needed for the recruitment and differentiation of osteoblast-forming mesenchymal stem cells (MSCs) [4], and furthermore drives the local revascularization needed for new bone formation [5]. Unfortunately, this process is dysregulated in comorbidities associated with a perturbed immune response (e.g. diabetes, increased age, rheumatoid arthritis, polytrauma) [6].

To date, macrophages have been established as the key target cells in the design of osteo-immunodulatory approaches for bone regenerative medicine [1, 4, 7]. This follows the premise that, although various innate and adaptive immune cell subsets can contribute to bone regeneration [8–10], macrophages have been identified as the sole immune players critical for bone formation. Bone fractures do not heal without involvement of macrophages [11], moreover macrophages are needed for bone induction by certain classes of bone-inductive materials [12, 13]. Important for effective immune modulation, macrophages are highly plastic cells, being able to adopt functionalities ranging from pro-inflammatory, anti-infective, immune-regulatory, or pro-regenerative, in response to the local stimuli [14]. Increasing evidence shows that the early crosstalk between inflammatory macrophages and MSCs determines the fate of bone regeneration. Initially, inflammatory macrophages are the source of a plethora of paracrine chemokines and cytokines (i.e., oncostatin M [OSM], tumor necrosis factor [TNF], stromal cell-derived factor-1 alpha [SDF-1α]) that promote the differentiation of MSCs into osteoblasts. In response, MSCs reciprocally regulate macrophage phenotype towards anti-inflammatory and pro-regenerative phenotype through prostaglandin E2 and inducible nitric oxide synthase (iNOS)-mediated immunosuppression [4, 15]. Currently, the most effective strategy to instruct the macrophage-MSC crosstalk and the subsequent osteogenic response is still unknown.

The targeting of pathogen recognition receptors in immune cells is an effective method of altering their behavior and the associated inflammatory responses [16, 17]. As such, even brief stimulation with microbial stimuli can lead to long-term alterations in immune cells [16]. Within this context, accumulating evidence has challenged the dogma that only lymphoid cells are privileged with immunological memory, but instead that myeloid cells also undergo long-lasting reprogramming after exposure to selective microbial stimuli [17]. This ancient immunological defense mechanism likely explains the immunological memory in biological systems lacking an adaptive immune system [18] or the non-specific immunity provided by live microorganism vaccines [19]. At the molecular level, this concept of ‘trained immunity’ is the result of well-characterized metabolic and epigenetic changes leading to chromatin modifications and altered susceptibility for transcription of inflammation-associated genes [20]. Accordingly, monocytes or their differentiated progeny are either sensitized (induction of training) or desensitized (induction of tolerance) depending on the primary stimulus. For example, Bacillus Calmette–Guérin (BCG) vaccine is a well-known ‘immune trainer’ [19, 21, 22], whereas bacterial lipopolysaccharide (LPS) is an archetypical inducer of ‘immune tolerance’ [23]. More recently, several changes in the bone marrow compartment were found to occur after immune training, such as enhanced myelopoiesis and subsequently increased altered responsiveness of circulating cells to secondary inflammatory cues [24, 25]. In vivo evidence shows that the metabolic and epigenetic changes in monocytes/macrophages following the withdrawal of a ‘training stimuli’ to persist for at least 3 months [21] and up to 1 year [26]. Therapeutic uses of innate immune memory currently are aimed at either enhancing immune responses for improved anti-microbial or anti-tumor responses, or preventing excessive immunopathologies such as auto-immune disorders [20].

To potentially broaden the clinical implications of trained immunity, this study is a first exploration of whether innate immune memory could have direct effects on osteogenic differentiation and osteoclast formation. Particularly, under resting and inflammatory conditions. We used an in vitro trained immunity protocol for monocyte-derived macrophages which has been able to reproduce in vivo training effects [21, 22]. We used common trainers (BCG, *Candida albicans*) and tolerizers (Pam3CSK4, LPS), known to induce opposing functional programs in macrophages.

## Materials and Methods

### Cell Isolation and Culture

Human tissues were obtained with the approval of the local medical ethical committee (University Medical Center [UMC] Utrecht) under the protocols METC 08–001/K and METC 07–125/C, and with the written informed consent of the participants.

For MSC isolation, bone marrow (*n* = 4 donors, mixed male/female) was harvested from the vertebrae of patients undergoing spinal surgery (UMC Utrecht). MSCs were isolated and frozen as described before [26]. Cells below passage 6 were used for the experiments. MSC expansion medium consisted of α-MEM (Invitrogen) with 10% heat-inactivated fetal bovine serum (FBS, Hyclone CSG0412, GE Healthcare Life Sciences), 100 U/mL penicillin and 100 U/mL streptomycin (Gibco), and 0.2 mM L-ascorbic-acid-2-phosphate (Sigma).

MSCs were characterized in terms of their cell surface marker expression profiles (MSC verification kit, FMC020, R&D Systems), following criteria defined by The International Society for Cellular Therapy [27]. Measurements on the BD FACSVerse flow cytometry system (Supplementary Fig. S1) confirmed the presence of CD90 (> 98%), CD73 (88–98%), CD105 (> 98%), and the absence of CD45, CD34, CD11b, CD79a and HLA-DR (all < 4%).

Monocytes were isolated from peripheral blood of healthy donors (mixed male and female, Mini Donor Service, UMC Utrecht) by CD14 magnetic-activated cell sorting (MACS®, Miltenyi Biotec), as described before [28]. Monocyte medium consisted of RPMI (Thermo Fisher Scientific) supplemented with 10% heat-inactivated FBS and 100 U/mL penicillin and 100 U/mL streptomycin. Cell cultures were always performed at 37 °C in a humidified atmosphere containing 5% CO_2_.

### Stimuli

Pam3CSK4 (PAM, Invivogen), lipopolysaccharide (LPS, from Escherichia coli O111:B4, Sigma), and *Bacillus Calmette–Guérin* (BCG, Medac, Lamepro B.V.) were purchased. *Candida albicans* (CA, ATCC 10231) was cultured to OD_660_ = 1.0 in malt agar medium. CA and BCG were gamma-irradiated at 10 kGy (Steris, Ede, the Netherlands) and stored at -80 °C in PBS with 40% (v/v) glycerol. The absence of viable microorganisms was confirmed by plate culture. The killed microbes were washed by centrifugation at 1200 × g for 10 min prior to use.

### Protocol for the Induction of Trained Immunity

The in vitro protocol to study innate immune memory response [29] consists of a ‘priming’ step (primary exposure with microbial stimulus), a resting period (no stimulus), and a ‘restimulation’ to challenge the primed-cells with either a strong inflammatory stimulus (such as LPS) or not-stimulated to mimic a physiological condition. Monocytes were seeded at 4.5 × 10^5^ cells/cm^2^ in flat-bottom wells in monocyte medium and allowed to rest for 48 h prior to stimulation (day 0). For cell priming, medium was supplemented with PAM (0.001, 0.1, 10 μg/ml), LPS (0.01, 1, 100 ng/ml), CA (10^2^, 10^4^, 10^6^ units/ml) or BCG (10^2^, 10^4^, 10^6^ units/ml). Medium with no stimulation was used as negative control. After 24 h, cells were washed twice with warm medium and differentiated for 6 days in macrophage medium, consisting of monocyte medium supplemented with 25 ng/ml recombinant human macrophage colony-stimulating factor (M-CSF, Peprotech). Afterwards, the medium was refreshed with monocyte medium and macrophages were given a secondary stimulation with either 50 ng/ml LPS (‘LPS restimulated’ groups) or with RPMI as a control (‘resting group’).

To investigate the pathways involved in the induction of innate immune memory in macrophages, monocytes were pre-incubated with the methyltransferase inhibitor 5′-methylthioadenosine (MTA, 1 mM, Sigma), the mTOR inhibitor Metformin (0.3 mM, R&D Systems), the histone demethylase inhibitor Pargyline hydrochloride (3 µM, Sigma), or the histone deacetylase inhibitor Trichostatin A (TSA, 50 nM, from Streptomyces sp., Sigma) for 1 h. The training immunity protocol was then carried out in presence of the inhibitors for an additional 24 h. Cells were washed twice with warm medium and differentiated as aforementioned.

### Macrophage CD Marker Expression Analysis

Macrophages were detached from the culture plate using cold 1 mM EDTA/PBS in combination with gentle cell scraping. A panel of CD markers was selected based on its utility to distinguish in vitro polarized macrophage subsets [31]. Cells were incubated for 30 min in cold staining solutions prepared by diluting the following fluorochrome-conjugated antibodies in 0.1% (w/v) bovine serum albumin and 1% (v/v) heat-inactivated human serum: CD11b-FITC (1:20, BioLegend, 101,205), CD16-FITC (1:20, BioLegend, 360,716), CD80-PE/Cy7 (1:20, BioLegend, 305,217), CD86-PE/Cy7 (1:50, BioLegend, 305,421), CD14-APC (1:20, BioLegend, 325,608), and CD163-APC (1:20, BioLegend, 333,609). Staining solution without antibodies and isotypes were used as control. Fluorescence was measured using the FACSVerse flow cytometer (BD) and analyzed using FlowJo (v.10.1., FlowJo LLC) after gating on the viable cell population in the FSC/SSC window. Values were expressed as the geometric mean fluorescence intensity (MFI) of the marker of interest over the MFI of the negative control.

### Osteoclast Differentiation

Monocytes were seeded at a density of 500,000 cells/cm^2^ in α-MEM supplemented with 10% heat-inactivated FBS and 100 U/mL penicillin and 100 U/mL streptomycin. Cells were allowed to rest for 48 h prior to stimulation. For the priming step, medium was supplemented with PAM (10 μg/ml) or BCG (10^6^ units/ml). Non-stimulated medium was used as negative control. After 24 h, cells were washed twice with warm medium and differentiated for 3 days in macrophage medium, consisting of monocyte medium supplemented with 25 ng/ml recombinant human M-CSF. Subsequently, osteoclast differentiation was induced with 25 ng/ml M-CSF and 50 ng/ml receptor activator of nuclear factor kappa-Β ligand (RANKL, Peprotech) for 5 days. Staining of tartrate-resistant acid phosphatase (TRAP) activity and osteoclast counting was performed as detailed before [28].

### Coculture Assay and Osteogenic Marker Analysis

To establish the macrophage-MSC cocultures, CD14^+^ cells were seeded at a density of 300,000 cells/cm^2^ in 96-well plates (alkaline phosphatase determination) or 48-well plates (calcium content determination), and subsequently primed and differentiated as aforementioned. MSCs were added in MSC expansion medium at 35,000 cells/cm^2^ and allowed to adhere for 24 h. MSC expansion medium was then replaced with osteoinductive medium, namely MSC expansion medium supplemented with 10 mM β-glycerophosphate (Sigma) and 10 nM dexamethasone (Sigma). Medium was refreshed every three days. Cocultures were either restimulated with LPS (‘LPS restimulated’ groups; to mimic inflammatory conditions) or with RPMI as a control (‘resting’ group; to mimic physiological conditions) during the first 72 h of osteogenic differentiation.

As an early marker of osteogenic differentiation, alkaline phosphatase (ALP) activity was determined at day 8 using a biochemical assay, based on the conversion of p-nitrophenyl phosphate (SigmaFast, Sigma) after lysis of the cells in 0.2% Triton X-100/PBS for 30 min. The absorbance was measured at 405 nm and corrected at 655 nm (Bio-Rad, Hercules). The ALP activity was normalized to the DNA content measured on the same lysate (Quant-It PicoGreen kit, Invitrogen). As a later marker of osteogenic differentiation, the calcium content was determined at day 17 after fixing the cells in 4% formaldehyde (w/v) and staining for 30 min with 0.2% (w/v) Alizarin Red S solution (pH 4.2, Sigma). For quantification, samples were washed five times with PBS, and treated with 10% cetylpyridinium to extract the calcium-bound Alizarin Red S. Absorbance was measured at 595 nm and corrected at 655 nm (Bio-Rad).

To visualize macrophages during coculture, the macrophages were labelled with DiL (Vybrant Multicolor Cell-Labeling Kit, Thermo Scientific) before adding to the MSCs. To visualize viable cells at the end of coculture, cells were stained with 2 µM calcein (LIVE/DEAD kit, Molecular Probes, Thermo Scientific). Fluorescence images were obtained with the Olympus IX53 fluorescence microscope.

### Metabolic Activity Assay

To determine the metabolic activity, cells were cultured for 90 min in medium supplemented with 10% (w/v) resazurin (Alfa Aesar, Thermo Fisher Scientific). Fluorescence from supernatant was measured using a 544 nm excitation filter and a 570 nm emission filter (Fluoroskan, Thermo Fisher Scientific). The background fluorescence signal provided by wells lacking cells was subtracted. The metabolic activity data were normalized to the DNA content (Quant-It PicoGreen kit, Invitrogen).

### Cytotoxicity Assay

Cytotoxicity was measured from the macrophages and MSC-macrophage cocultures stimulated with LPS for 24 h (*n* = 6). Cytotoxicity of the different culture conditions were determined from the supernatants using a lactate dehydrogenase enzyme (LDH) assay kit (CyQUANT™ LDH Cytotoxicity Assay Kit, Invitrogen) according to the manufacturer’s instructions.

### Enzyme-Linked Immunosorbent Assay (ELISA) Analysis

Measurement of cytokine levels by ELISA was conducted on primed macrophage cultures (after 24 h) and MSC-macrophage coculture stimulations (after 72 h). Supernatants were collected and stored at − 20 °C for cytokine measurements. The concentrations of TNF, IL-6, OSM, SDF-1α (human DuoSet® kits, R&D Systems), and bone morphogenetic protein 2 (BMP-2, BGK8C060, Peprotech) were measured using ELISA kits, according to the manufacturer’s instructions. Culture consisting of non-primed macrophages were used as control.

### Phagocytosis Assay

Primed/differentiated macrophages were pre-labeled with DiO (Vybrant Multicolor Cell-Labeling Kit, Thermo Scientific). *Staphylococcus aureus* (*S. aureus*) were cultured and gamma-irradiated as described before [30]. Bacteria were fluorescently labeled by incubation in a 25 μg/ml propidium iodide (Thermo Fischer Scientific) solution (in deionized water) for 1 h at room temperature in the dark and subsequently opsonized in human pooled serum for 30 min at 37 °C. The labeled/opsonized bacteria were resuspended in monocyte medium and added to the macrophages (MOI of 1:50, macrophages:bacteria). After 2 h incubation at 37 °C, samples were washed three times with PBS and fixed in 4% formaldehyde (w/v) for 15 min at room temperature. Images for the DiO and PI signals were obtained with the Olympus IX53 fluorescence microscope.

The percentage of DiO-positive cells were determined with CellProfiler (version 4.0.7) using the following pipeline: 1) images were converted into grayscale images using the ‘ColorToGray’ function. 2) noise was removed using the ‘ReduceNoise’ (Size: 100, Distance: 2, Cut-off distance: 0.1) and ‘EnhanceOrSuppressFeatures’ modules (Feature type: Speckles, size: 150). 3) cells were identified using the ‘IdentifyPrimaryObjects’ module (Size: 100–700, Threshold strategy: Global, Thresholding method: Minimum Cross-Entropy, Threshold correction factor: 0.1). 4) the ‘RescaleIntensity’ module was used to enhance the signal while maintaining the differences in intensity between each cell. 5) the ‘IdentifyPrimaryObjects’ module was used to identify bacteria (Size: 50–700, Threshold strategy: Global, Thresholding method: Otsu two-classes, Threshold correction factor: 1, Threshold smoothing scale: 10). 5) the ‘RelateObjects’ function was used to identify the percentage of positive cells.

### Statistics

All data are presented as mean ± SEM, with the group sizes indicated in the figure legends. Statistical analysis was performed in SPSS (v.25, IBM). Differences in CD marker expression were analyzed using a linear mixed model approach. All other data were analyzed using the Wilcoxon signed-rank test. *p* < 0.05 was used as a threshold for significance.

## Results

### Macrophage Changes following the Trained Immunity Model

**Fig. 1:**
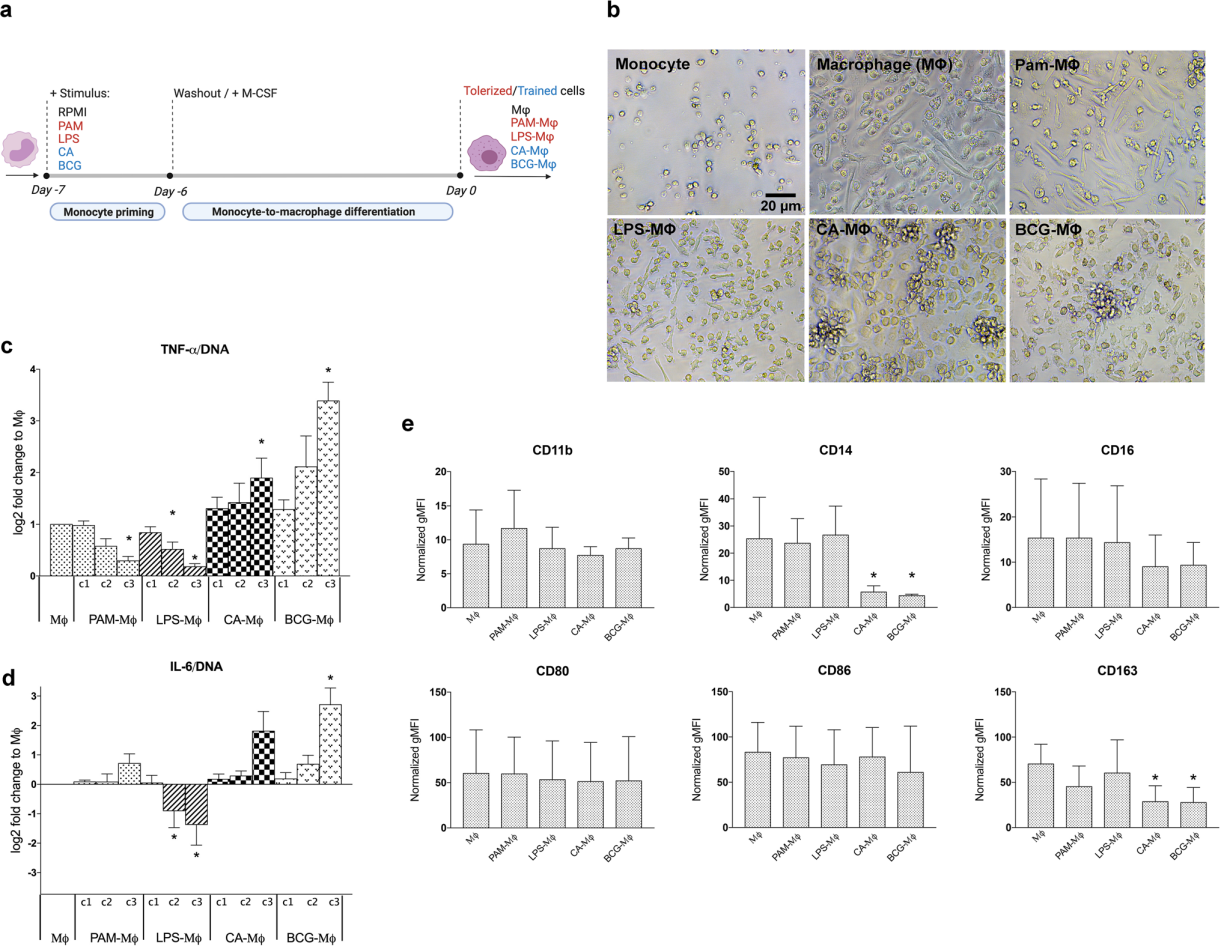
**(a)** In vitro model to induce trained or tolerant macrophages. Monocytes were exposed for 24 h with one of the stimuli shown or with RPMI (negative control). After the stimulus was washed away, the cells were differentiated for 6 days. Cells were characterized or restimulated with LPS for cytokine measurement. (**b)** Morphology of undifferentiated monocytes after 7 days, in comparison to monocytes primed with PAM (10 μg/ml), LPS (100 ng/ml), CA (10^6^ units/ml) or BCG (10^6^ units/ml) and differentiated into macrophages (Μφ) for 6 days. (**c,d)** TNF **(c)** or IL-6 **(d)** production by macrophages was normalized for the DNA content after restimulation with LPS for 24 h. c1-c3 denote the increasing concentrations of PAM (0.001, 0.1, 10 μg/ml), LPS (0.01, 1, 100 ng/ml), CA (10^2^, 10^4^, 10^6^ units/ml) and BCG (10^2^, 10^4^, 10^6^ units/ml) used during the priming step (mean ± SEM, *n* = 6). Non-primed macrophages (Μφ) served as control. **(e)** Flow cytometric analysis of CD marker expression on macrophages after 24 h of priming with PAM (10 μg/ml), LPS (100 ng/ml), CA (10^6^ units/ml) or BCG (10.^6^ units/ml) and 6 days differentiation. Non-primed macrophages (Μφ) served as control. Data were normalized to the values of unstained cells (mean ± SEM, *n* = 3). *p < 0.05 Wilcoxon signed-rank test (panel c,d) or linear mixed model (panel e)

Based on optimal conditions from literature, the exposure of cells to PAM and LPS leads to a tolerant state while CA and BCG leads to a trained state [22, 29]. Cells were exposed to the different agents for 24 h, followed by washing and differentiation of the cells for 6 days in the absence of microbial stimuli (Fig. 1a). Thereafter, cells were either restimulated with LPS for cytokine induction and/or studied for their osteo-modulatory activity in cocultures with MSCs.

Following the in vitro protocol, M-CSF differentiated macrophages (Μφ) consisted of a mixed cell population with round or elongated spindle-shaped morphologies (Fig. 1b). Priming with PAM (PAM-Μφ) led to a predominantly elongated cell morphology, whereas LPS-primed cells (LPS-φ) were mostly rounded. Priming with CA (CA-Μφ) or BCG (BCG-Μφ) showed an intermediate phenotype and promoted cell clustering.

There were no differences in cytokine production between primed and non-primed macrophages under resting conditions (not shown). However, restimulation of the macrophages with LPS changed the dynamics of cytokine production. PAM-Μφ showed a decreased in TNF production (-70%, *p* = 0.028) as compared to Μφ (Fig. 1c). While LPS-Μφ showed decreased production of both TNF (-80%, *p* = 0.028) and IL-6 (-60%, *p* = 0.043) (Fig. 1c, 1d). This aligns with the characteristics of a tolerized immune phenotype. In contrast, CA-Μφ was associated with increased production of TNF (1.9-fold, *p* = 0.028). While BCG-Μφ enhanced production of TNF (3.4-fold, *p* = 0.028) and IL-6 (6.5-fold, *p* = 0.046) (Fig. 1c, 1d), clearly depicting a trained phenotype. The effects of innate immune memory were seen to be dose-dependent and the contrasting features of tolerized and trained phenotype were most prominent for the highest concentration of the stimulus tested. Therefore, for the remainder of the experiments, macrophages were primed using the highest concentration of microbial stimuli.

To determine whether priming of macrophages affect polarization towards a classic (M1) or alternative (M2) phenotype, several phenotypic markers were evaluated [31]. Macrophage differentiation markers such as CD11b, CD16 and CD14 were measured. Including CD80/CD86 as markers of classic (M1) and CD163 for alternative (M2) macrophage phenotype. Our data showed that the priming step did not affect surface expression of macrophage differentiation markers CD11b and CD16. However, CA-Μφ and BCG-Μφ had a significant decrease in their CD14 (*p* < 0.008). Priming also did not affect expression of CD80/CD86 markers. For CD163, CA-Μφ and BCG-Μφ showed a decrease compared to Μφ (< 0.029) (Fig. 1e).

### Effect of the Induction of Innate Immune Memory with Macrophage-MSC Cross Talk and Osteogenesis

**Fig. 2:**
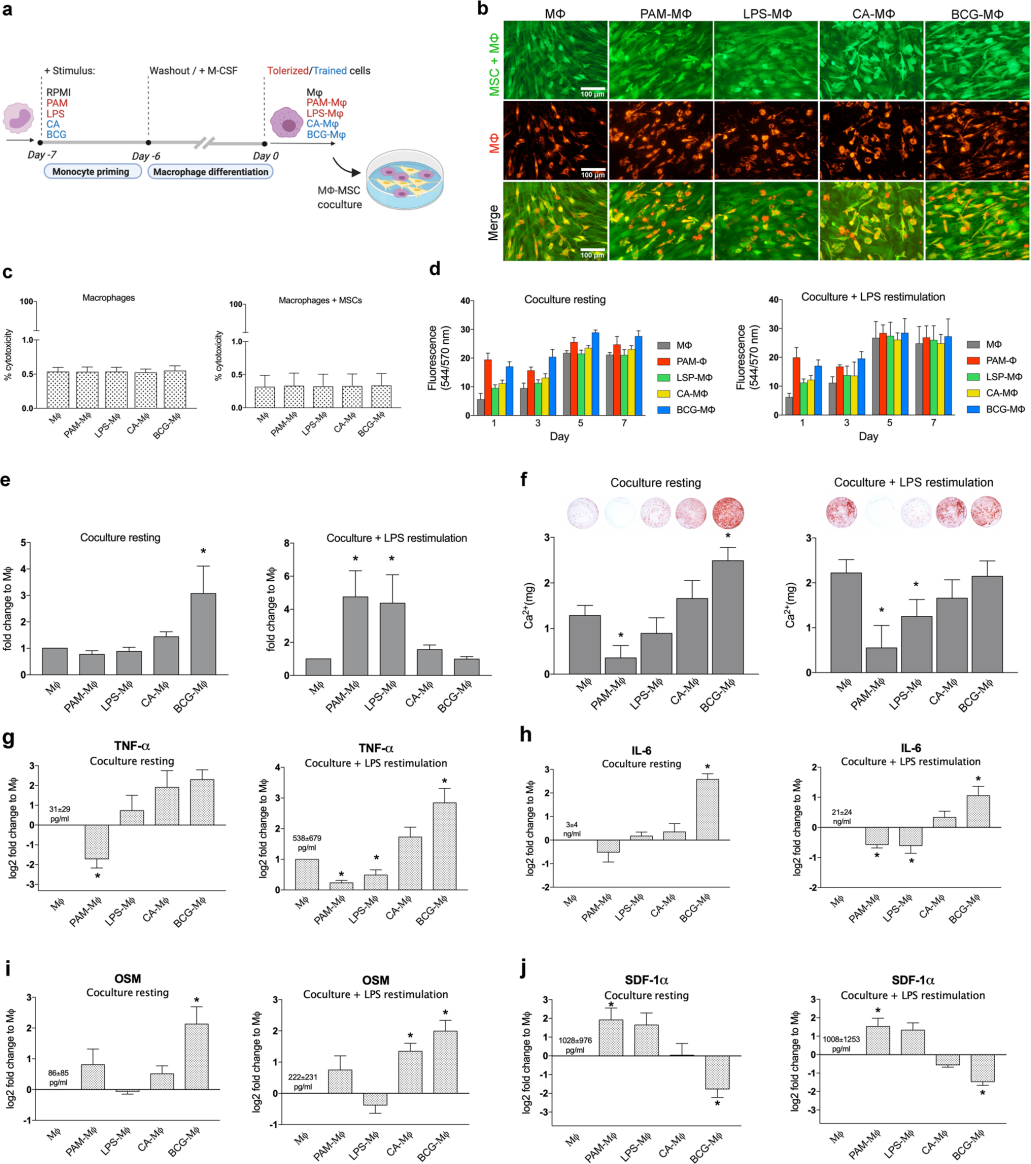
In vitro MSC-macrophage coculture model. (**a)** Monocytes were exposed for 24 h to PAM (10 μg/ml), LPS (100 ng/ml), CA (10^6^ units/ml), BCG (10^6^ units/ml), or with RPMI (negative control). After the stimulus was washed away, the cells were differentiated for 6 days and cocultured with MSCs. (**b)** Fluorescence images showing stained MSCs/macrophages (calcein-AM, green), prelabeled macrophages (DiL, red) and the merged channels after 21 days of coculture. (**c)** Cytotoxicity according to the LDH assay. LDH was measured in the supernatants of macrophages and cocultures stimulated with LPS for 24 h (mean ± SEM, *n* = 6). (**d)** Total metabolic activity in MSC-macrophage cocultures at different timepoints. Cocultures were either restimulated with LPS (LPS restimulation) or not (resting) during the first 72 h of coculture (mean ± SEM, *n* = 4). **(e)** Day 8 ALP activity normalized for the DNA content in cocultures either (LPS restimulation) or not (resting) stimulated with LPS during the first 72 h of coculture (mean ± SEM, *n* = 7). (**f)** Day 17 calcium content in cocultures either with LPS restimulation or resting condition during the first 72 h of coculture (mean ± SEM, *n* = 5). (**g-j)** TNF **(g)**, IL-6 **(h),** OSM **(i)** or SDF-1α** (j)** levels in MSC-macrophage cocultures as measured in the supernatants collected after 72 h. Cocultures were either stimulated with LPS (LPS restimulation) or not (resting) during the first 72 h of coculture (mean ± SEM, *n* = 7). *p < 0.05 Wilcoxon signed-rank test

Macrophages are known to have pro-osteogenic effects on MSCs, whereby the collective evidence suggests that these effect are mediated by soluble mediators, including various pro-inflammatory cytokines [4]. Hence, it was investigated whether the opposing cytokine expressions, including TNF and IL-6 (Fig. 1c, 1d), in the trained and tolerized macrophages would lead to different osteogenic responses in MSCs. MSC-macrophage cocultures were either restimulated with LPS, mimicking a pro-inflammatory condition, or not stimulated, mimicking a physiological condition, during the initial 72 h, denoted as the ‘LPS restimulation’ and ‘resting’ groups, respectively (Fig. 2a) [4, 15].

The validity of the coculture model was first assessed. Macrophages remained stable in the coculture up to 21 days (Supplementary Fig. S2). Fluorescent staining of the coculture depict confluency of MSCs at day 21 and no difference was observed in macrophage viability or distribution between the different groups (Fig. 2b). Furthermore, no change in cytotoxicity level was detected via an LDH release assay in the cocultures when compared to macrophage alone, irrespective of its primed status (Fig. 2c). The cocultures showed an increased trend in total metabolic activity over time, with or without LPS restimulation, in which a plateau was detected at day 5 (Fig. 2d).

As an early marker associated with osteogenic differentiation capacity of human MSCs [35], the ALP activity was assessed after 8 days of coculture. Under resting conditions, a threefold increase (*p* = 0.018) in ALP activity normalized to DNA content was found in MSC + BCG-Μφ as compared to MSC + Μφ cocultures (Fig. 2e). Following LPS restimulation, however, at least a fourfold higher ALP activity/DNA was found in the tolerized PAM-Μφ (4.7-fold, *p* = 0.018) or LPS-Μφ (4.4-fold, *p* = 0.018) cocultures compared to Μφ, whereas for the trained CA-Μφ and BCG-Μφ, the ALP activity/DNA of the MSCs did not change (Fig. 2e).

Late osteogenic differentiation was evaluated in terms of the amount of calcium deposition in both the resting and restimulation cocultures. At day 17, calcium deposition was seen in the MSC-Μφ groups (Fig. 2f), whereas, in contrast, no calcium deposition was seen in MSC monocultures at this time point (not shown). The effect of macrophages on mineralization was dependent on their trained or tolerized state, and whether or not inflammatory conditions were applied. Under resting conditions, PAM-Μφ medium decreased the matrix mineralization of the MSCs with 70% (*p* = 0.043), with complete inhibition of mineralization in 3 out of 5 donors, whereas medium from BCG-Μφ macrophages increased the matrix mineralization of the MSCs by twofold (*p* = 0.043). Under pro-inflammatory conditions (LPS restimulation), PAM-Μφ (-75%, *P* = 0.043) and LPS-Μφ (-40%, *p *= 0.043) decreased mineralization activity whereas CA-Μφ and BCG-Μφ did not further enhance osteogenic differentiation compared to the control (Fig. 2f).

The levels of soluble factors known to be important in the MSC-macrophage osteogenic cross talk [4, 32, 33] were measured in the culture supernatant. In general, similar trends were observed in resting and LPS-restimulated cocultures for each of the factors, despite large differences in their baseline levels. Furthermore, cocultures comprising PAM-Μφ and BCG-Μφ macrophages generally showed opposing cytokine profiles. TNF (Fig. 2g), IL-6 (Fig. 2h), and OSM (Fig. 2i) levels were significantly higher, and SDF1-α expression (Fig. 2j) was significantly lower, in cocultures comprising BCG-trained macrophages relative to cocultures comprising non-primed macrophages. In cocultures comprising PAM-Μφ, TNF and IL-6 levels were significantly lower, while SDF1-α expression (Fig. 2j) was significantly higher, relative to cocultures comprising Μφ without induction of innate immune memory. Levels of BMP-2, a potent osteoinductor, were below the detection limit of the assay (31.2 pg/ml).

### Functional, Metabolic, and Epigenetic Changes in PAM-Μφ and BCG-Μφ

**Fig. 3:**
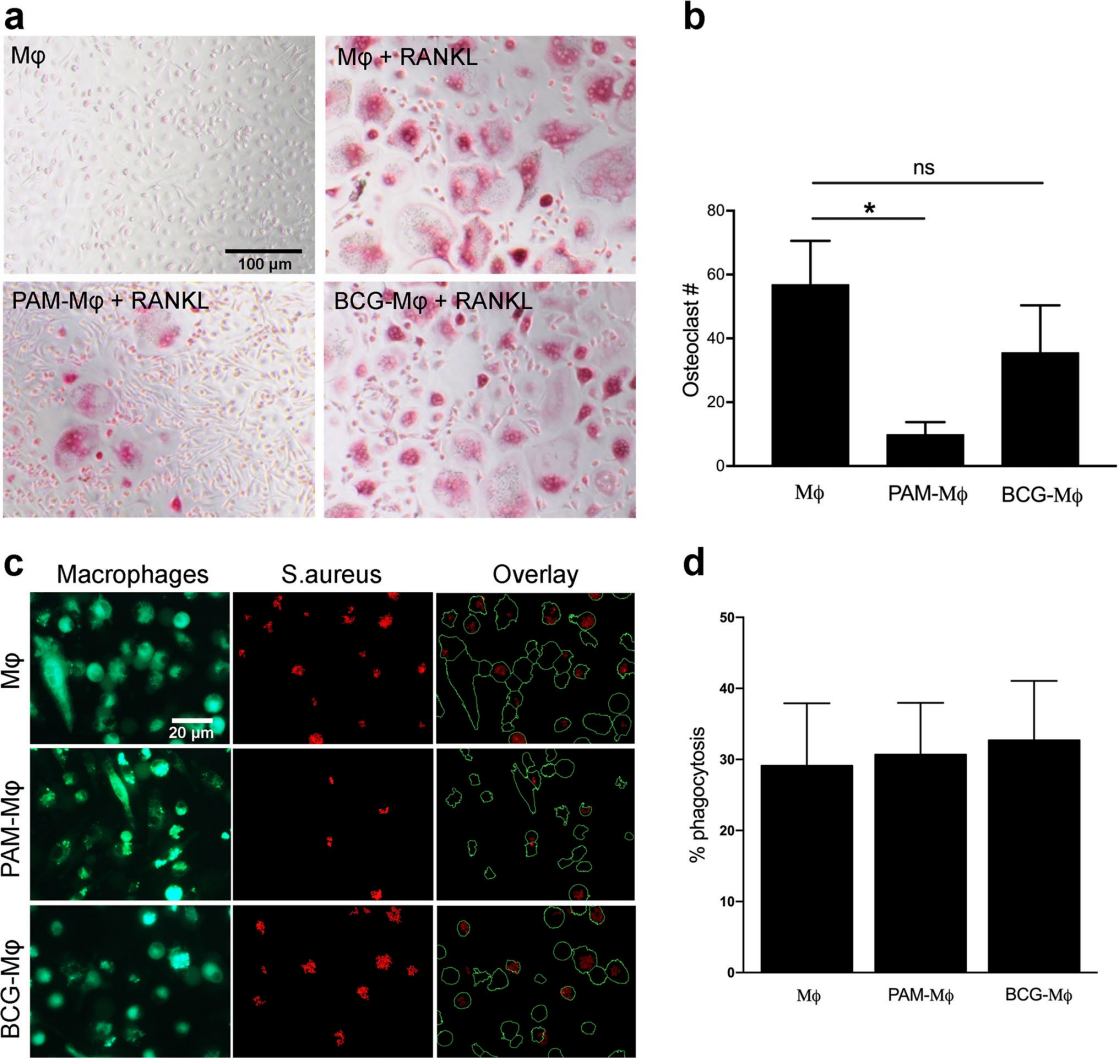
Osteoclast and phagocytic activity in primed cells. **(a)** TRAP staining showing the presence of osteoclasts. Monocytes were exposed to PAM (10 μg/ml), BCG (10^6^ units/ml), or with RPMI (negative control) for 24. After the stimulus was washed away, the cells were differentiated for 3 days in the presence of M-CSF, and another 5 days in the presence of M-CSF and RANKL. No osteoclast formation was seen in the absence of RANKL. **(b)** Quantification of the number of osteoclasts (mean ± SEM, *n* = 7). **(c)** Fluorescence images showing DiO-prelabeled macrophages (green) and their phagocytosis of PI-labeled killed S. aureus (red) after 2 h incubation. The overlays show the result of the CellProfiler pipeline used to quantify % phagocytosis. **(d)** Quantification of the bacterial phagocytosis (mean ± SEM, *n* = 3). *p < 0.05 Wilcoxon signed-rank test

Considering that the PAM-Μφ or BCG-Μφ subsets showed most prominent and opposing osteo-modulatory activity (Fig. 2), we further investigated any changes in macrophage behavior that is relevant for bone metabolism. First, the ability of Mφ, PAM-Μφ, and BCG-Μφ to differentiate into osteoclasts was evaluated. Stimulation with RANKL induced the formation of osteoclasts in all three groups (Fig. 3a). PAM-Μφ had reduced osteoclastic differentiation capacity in presence of RANKL (-80%, *p* = 0.028), whereas this was not significantly affected in BCG-Μφ, compared to non-primed macrophages (Mφ) (Fig. 3b). Second, it was found that the induction of neither training nor tolerance affected the macrophages’ normal ability to phagocytose *S. aureus* (Fig. 3c, d). This shows that trained or tolerized macrophages may have unaltered anti-infective responses when confronted with bacterial strains that have a high tendency to colonize biomaterials [34]*.*

**Fig. 4:**
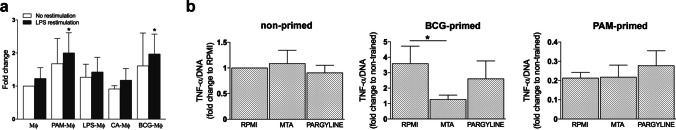
Metabolic/epigenetic changes in macrophages during the priming step. **(a)** Metabolic activity in macrophages normalized for their DNA content. Monocytes were exposed for 24 h with PAM (10 μg/ml), LPS (100 ng/ml), CA (10^6^ units/ml), BCG (10^6^ units/ml), or with RPMI (negative control). After the stimulus was washed away, the cells were differentiated for 6 days. The metabolic activity was measured after 24 h, either in absence or presence of LPS (mean ± SEM, *n* = 3). **(b)** TNF production normalized to DNA after 24 h LPS restimulation in non-primed, PAM-primed (10 μg/ml) or BCG-primed (10^6^ units/ml) cells in the absence or presence of the histone methyltransferase inhibitor MTA or the histone demethylase inhibitor pargyline (mean ± SEM, *n* = 5–7). *p < 0.05 Wilcoxon signed-rank test

The functional reprogramming of myeloid cells by exposure to inducers of trained immunity is known to have both a metabolic and epigenetic basis [20]. Both PAM-Μφ (+ 62%, *p* = 0.043) and BCG-Μφ (+ 60%, *p* = 0.043) had an increased metabolic activity after LPS restimulation when compared to their respective non-primed macrophages (Fig. 4a). The inhibition of histone methyltransferases with 5′-deoxy-5′-methylthioadenosine (MTA) or histone demethylases with pargyline did not affect the cytokine expression in non-primed cells (Fig. 4b). In BCG-Μφ, MTA reduced the training effect of BCG as shown by the decreased TNF production (-64%, *p* = 0.046), whereas pargyline did not significantly alter the TNF expression. No effects of MTA or pargyline were seen in PAM-Μφ. A possible cytotoxic effect of the inhibitors was excluded as shown by the cells’ unchanged LDH release (Supplementary Fig. S3).

## Discussion

Macrophages are cells of the innate immune system that plays a role in almost every aspect of the biological system, both in homeostatic and repair process. While carrying out their function during repair and regeneration, macrophages engage in active interactions with MSCs. Both cells influencing the behavior of the other [4, 35]. In this study we explore the effect of trained immunity induction of macrophages on MSCs ability to undergo osteoblast differentiation and macrophage’s ability to differentiate into osteoclast. Of the stimuli investigated as inducers of trained immunity, PAM and BCG had the strongest effect on altering macrophage’s osteogenic-stimulating phenotype by dampening or boosting the inflammatory milieu. Moreover, the changes in macrophage’s functional state due to exposure by PAM (tolerant state) or by BCG (trained state) led to opposing results in their capacity to differentiate into osteoclasts.

The experimental protocol, including exposure to the stimuli (24 h) and resting (6 days) intervals, was based on work done by others, describing the optimal conditions for the induction of training and tolerance in human monocytes [29]. The length of the resting period is strongly associated with the magnitude of cytokine responses, and is likely related to the time required for epigenetic changes to occur that are involved in innate immune memory [22, 28, 29]. For detailed description on the metabolic and epigenetic changes that occur following the experimental protocol please refer to Arts et.al. (2016) [36]. In this study, Macrophages were generated using M-CSF, as opposed to the macrophage differentiation factors GM-CSF or IFN-γ, to avoid the strong polarization of cells into an M1 or M2 phenotype. Therefore resemble a broader spectrum of tissue-resident and inflammatory macrophages that can contribute to bone formation [22, 37, 38]. Although this protocol yielded cells with distinct pro-osteogenic and pro-inflammatory signatures, the osteogenic-stimulating responses may still be even further increased by optimizing the exposure and resting period or by applying different stimulus.

Together, the data show that the in vitro protocol for induction of innate immune memory in macrophages established cells with different morphological, functional, and phenotypic characteristics depending on the stimulus applied. Pro-inflammatory cytokine levels returned to basal levels after removal of the initial stimulus. This suggests an unchanged functional state of these cells, typical for both ‘trained’ or ‘tolerant’ cells. However, changes in morphological characteristics and phenotypic expressions were observed, indicative of (partial) differentiation of cells. Accordingly, the training agents (CA and BCG) seemed to impact macrophage phenotype more than the tolerizing agents (PAM and LPS), as shown by the changes in their CD marker expression profile. A decreased expression of the CD163 marker (M2 phenotype) was observed in CA-Μφ and BCG-Μφ, suggestive of a more pro-inflammatory M1 phenotype, however, this was not accompanied by an increased expression of other M1 markers such as CD80/CD86 [31]. In addition, exposure of macrophages with the different stimuli did not alter their phagocytic activity [39]. Taken together, CA-Μφ and BCG-Μφ- trained cells appear as functionally distinct cells, which cannot be identified using the simplified M1/M2 classification, in accordance with other reports [40, 41]. As to date, the relative importance of M1 and M2-derived phenotypes in promoting bone formation has furthermore been challenging to pinpoint [4], the trained or tolerized status of macrophages may be a better predictor of this behavior.

We found that epigenetic changes could play, at least in part, a role in the pro-osteogenic phenotype of BCG-Μφ. First, inhibition of histone methyltransferases with MTA reduced the BCG training effect, even though the inhibitors of histone deacetylase or histone demethylases did not significantly influence the training effects of BCG. Second, BCG-Μφ showed an increased metabolic state, and it is known that metabolic shifts are strongly linked to the epigenetic landscape [17]. Since monocyte-to-macrophage differentiation is also driven by epigenetic changes [22, 40], more in-depth analysis is needed to unravel to what extent epigenetic mechanisms are involved, and how different training and differentiation markers are affected.

Of note, by definition, innate immune memory in macrophages is characterized by their heightened or mitigated responses only following stimulation to a secondary agent, as the cells are allowed to return to a functional steady state after the initial exposure [16]. Although this was clearly the case in macrophage monocultures, surprisingly, the changes in (osteo)-immunomodulatory effects of trained (BCG-Μφ) and tolerized cells (PAM-Μφ) were apparent in the cocultures without a second stimulation. Since MSCs and macrophages have a bidirectional interaction in cocultures [4, 42, 43], it can be speculated that the presence of MSCs can act as a secondary stimulus for primed macrophages.

As reviewed by Pajarinen et al. [4], macrophages secrete baseline levels of cytokines and growth factors that promote the osteogenic differentiation of MSCs. These pro-osteogenic effects are predominantly mediated by soluble factors, whereas cell–cell interactions play only a minor role. Here, we show that BCG-Μφ improved osteogenic differentiation of MSCs compared to non-stimulated macrophages, in MSC coculture. Conversely PAM- Μφ inhibited the process. One possible explanation for these opposing results is a difference in cytokine secretion. TNF and IL-6 are among the mediators known to be regulators in bone regeneration in vivo [44, 45] and were upregulated in MSC-BCG-Μφ cocultures. While, PAM-Μφ macrophages suppressed pro-inflammatory cytokine production in cocultures, which likely resulted in impaired osteoblast differentiation. In addition, this study is the first to show a role for OSM upregulation in trained innate immunity. OSM has an increasingly appreciated role in stem cell osteogenic differentiation [4, 33, 46], and it is of interest to determine to what extent the COX-2/PGE2 pathway is involved in the pro-osteogenic activity of BCG-Μφ [47, 48].

This study found that PAM-Μφ macrophages largely lost their ability to differentiate into osteoclasts. Although the mechanism is still unknown, we hypothesize that it may involve changes in the expression of receptor activator of nuclear factor kappa-Β (RANK) and the cells’ responsiveness towards RANKL stimulation. This argument is supported by the observation that certain pathogen recognition receptors are downregulated in tolerized macrophages which reduce their sensitivity to stimuli [23, 49]. This phenomena could have a negative impact for bone regenerative strategies, as it can hamper new bone formation around bone-inducing biomaterials [12] or bone remodeling [3]. Thus, tolerized macrophages may display an unfavorable phenotype for bone formation under physiological conditions.

Immune therapies are ideally tailored to account for the heterogeneity in patients’ immune statuses [50, 51]. Since trained and tolerized macrophages have opposing osteo-immunomodulatory activities, the appropriate immune activation or suppression by these cells may require fine-tuning to the tissue demand, i.e., to compensate for physiological or elevated inflammation. This is important in the context of fracture healing, since the inflammatory milieu of the fracture hematoma is critical for repair. On the one hand, an enhanced inflammatory response is more likely beneficial under physiological conditions or when the normal inflammatory response is impaired due to comorbidities or anti-inflammatory drugs [6, 52, 53]. On the other hand, harmful hyperinflammatory conditions could be dampened by induction of tolerance to restore bone regeneration or prevent chronic bone loss [41, 54]. In addition, induction of tolerance may benefit patients with polytrauma [55] or implant wear particle-induced disease [56]. Moreover, our findings suggest that under enhanced inflammatory conditions (i.e., LPS restimulation), tolerized macrophages restored the ALP activity and early osteoblast differentiation, while trained macrophages lost their stimulatory effect on early osteoblast differentiation.

Innate immune memory has been particularly well-established in monocytes/macrophages, nevertheless, other (immune) cell types may also play a role in improved bone healing responses. For example, the activation status of neutrophils is correlated with the outcome of bone healing [55, 57, 58], and BCG vaccination recently was shown to cause functional reprogramming of neutrophils by induction of innate immune memory [59]. In fact, innate immune memory may even exist in resident cells with immune-modulating properties, such as MSCs [60]. Thus, there is a need for future explorations on the role of innate immune memory has on bone physiology and repair.

In the current study, we investigated a limited number of primary stimuli. In the future, this panel could be expanded with other known innate immune trainers, such as endogenous inducers of innate immunity (e.g. oxidized low-density lipoprotein particles [19] or certain biomaterials, e.g. graphene or gold nanoparticles [61, 62].

Further study is required to more firmly pinpoint the soluble factors that mediates the osteo-immunomodulatory effect of trained or tolerized cells. Moreover, in vivo training strategies [21, 41] can be followed to study their effect on bone tissue regeneration. In addition to the observed in vitro effects, trained macrophages may have additional osteo-immunomodulatory effects in vivo, which exceed their effects on MSC differentiation. These at least include the modulation of tissue vascularization [5] or inducing activity of specific T cell subsets, beneficial for bone healing, including IL-17-producing T cells [10, 41, 63].

## Conclusion

Macrophages and MSCs play an important role in bone regeneration, making their interactions a critical aspect to consider in the development of effective approaches for bone regenerative medicine. The direct effect of macrophages’ immunological memory has on bone forming and bone resorbing cells should be taken into account when designing personalized immune-based therapeutic interventions, since patient’s immunological status may influence treatment outcome.

### Supplementary Information

Below is the link to the electronic supplementary material.

Supplementary file1 (PDF 370 KB)

## Data Availability

All data generated during this study are included in this published article and its supplementary information files.

## Abbreviations

***Μφ***: Macrophages
***MSCs***: Mesenchymal stem cells
***BCG***: Bacillus Calmette–Guérin
***CA***: *Candida albicans*
***PAM***: Pam3CSk4
***LPS***: Lipopolysaccharide
***TNF***: Tumor necrosis factor
***IL-6***: Interleukin-6
***OSM***: Oncostatin M
***SDF-1α***: Stromal cell-derived factor-1 alpha (also known as CXCL12)
***CD***: Cluster of differentiation
***HLA-DR***: Human Leukocyte Antigen – DR isotype
***ALP***: Alkaline phosphatase
***DNA***: Deoxyribonucleic acid
***BMP-2***: Bone morphogenetic protein 2
***M-CSF***: Macrophage colony-stimulating factor
***RANKL***: Receptor activator of nuclear factor kappa-Β ligand
***RANK***: Receptor activator of nuclear factor kappa-Β
***TRAP***: Tartrate-resistant acid phosphatase
***LDH***: Lactate dehydrogenase enzyme
***MTA***: Methylthioadenosine
