# Supplementary material for: Trained innate immunity modulates osteoblast and osteoclast differentiation
Source: Stem Cell Rev Rep. 2024 Mar 13;20(4):1121–34. doi: 10.1007/s12015-024-10711-9 (PMC11087362; doi:10.1007/s12015-024-10711-9)
Supplement: Supplementary file 1 — Supplementary file1 (PDF 370 KB) [file 12015_2024_10711_MOESM1_ESM.pdf]

Supplementary data

Trained innate immunity modulates osteoblast and osteoclast differentiation

N.R. Rahmani[a*], R. Belluomo[a], M.C. Kruyt[a,b], D. Gawlitta[c], L.A.B. Joosten[d,e], H. Weinans[a,f], M. Croes[a]

[a] Department of Orthopedics, University Medical Center Utrecht, Utrecht, the Netherlands

[b]Department of Developmental Biomedical Engineering, Twente University, the Netherlands

[c]Department of Oral and Maxillofacial Surgery, Prosthodontics and Special Dental Care, University Medical Center Utrecht, Utrecht, the Netherlands

[d]Department of Internal Medicine, Radboudumc, Nijmegen, The Netherlands

[e]Department of Medical Genetics, Iuliu Hatieganu University of Medicine and Pharmacy, Cluj-Napoca, Romania

[f]Department of Developmental Biomechanical Engineering, TU Delft, the Netherlands

Supplementary Figure 1

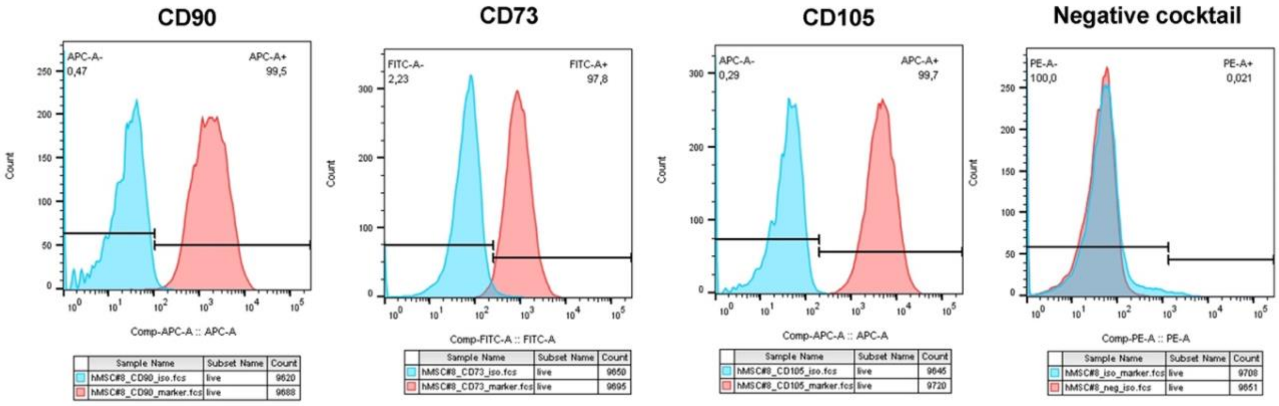

MSC CD marker expression. MSCs were stained with specific antibodies (red histograms) or with the isotype controls (blue histograms). The negative cocktail consists of antibodies specific for CD45, CD34, CD11b, CD79a and HLA-DR. The histograms show the result for one, representative, MSC donor.

Supplementary Figure 2

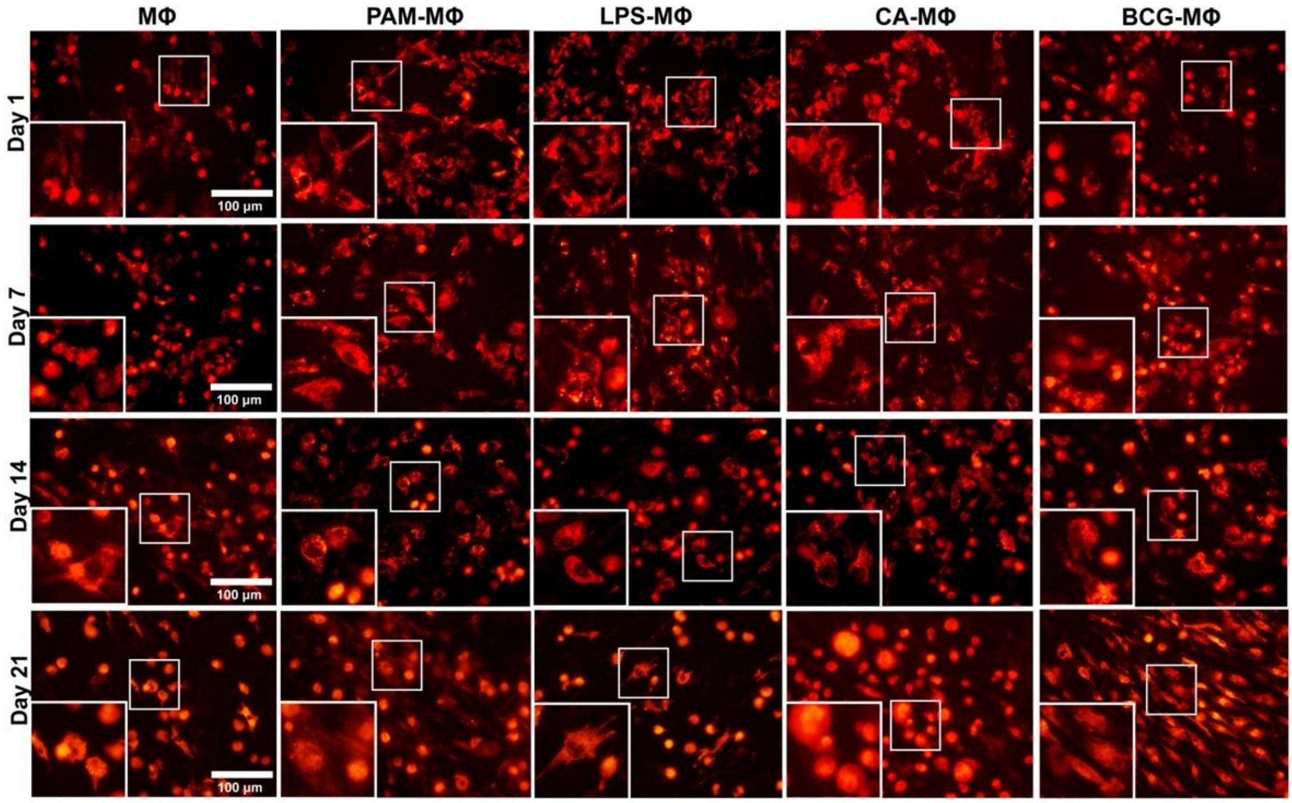

Macrophage tracking in co-culture. Differently primed macrophages were labeled with DiO and brought into coculture with MSCs. Images show DiO-prelabeled macrophages at different times of the coculture.

Supplementary Figure 3

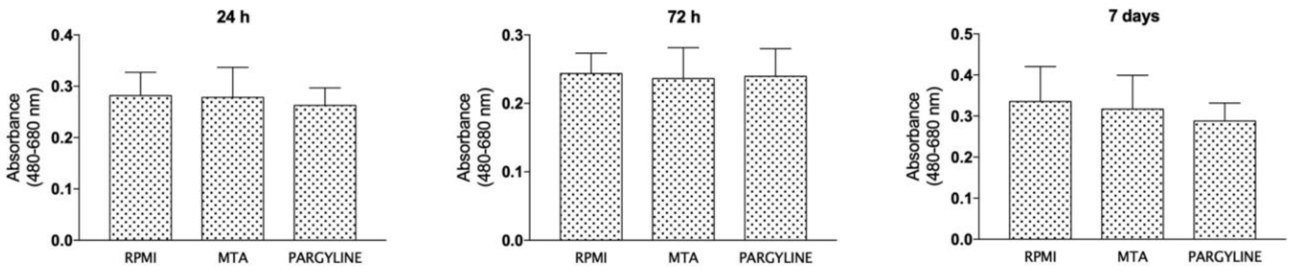

Effect of inhibitors on cell viability. Monocytes were stimulated for 24 hours with either culture medium, MTA (1 mM) or pargyline (3 µM). After washing, cells were differentiated with M-CSF. At the indicated time points, supernatants were collected and subjected to the LDH cytotoxicity assay. Data are presented as mean ± SEM (n = 5).
